# The Rate of Postoperative Complications in Complicated Acute, and Subacute Type B Aortic Dissections after TEVAR vs. PETTICOAT Procedures: Systematic Review and Meta-Analysis

**DOI:** 10.3390/medicina59122150

**Published:** 2023-12-12

**Authors:** Natalia Niklas, Klaudia Królikowska, Kinga Zdrodowska, Piotr Gutowski, Arkadiusz Kazimierczak, Paweł Rynio

**Affiliations:** Department of Vascular Surgery, Pomeranian Medical University in Szczecin, Al. Powstańców Wielkopolskich 72, 70-111 Szczecin, Poland; nniklas488@gmail.com (N.N.); klaudia.1799@wp.pl (K.K.); kinga.zdrodowska1@gmail.com (K.Z.); piotr_gutowski@poczta.onet.pl (P.G.); biker2000@wp.pl (A.K.)

**Keywords:** PETTICOAT, TEVAR with bare metal stent, endovascular reinterventions

## Abstract

*Background and Objectives*: Provisional extension to induce complete attachment (PETTICOAT) is suggested as being associated with a lower incidence of aorta-related events and fewer reinterventions compared to thoracic endovascular aortic repair (TEVAR) in patients with complicated acute, and subacute type B aortic dissections. *Materials and Methods*: This article is a systematic review and meta-analysis following the PRISMA guidelines. The Medline, PubMed, Embase, and Cochrane databases were searched, starting on 21 February 2022 and ending on 22 June 2022, to identify studies that investigated the rate of postoperative complications in patients treated with the PETTICOAT compared to the TEVAR. A random effects meta-analysis was performed. Of 2350 studies, 5 studies involving 360 patients were included: 143 patients after the PETTICOAT procedure and 217 after the TEVAR. *Results*: The meta-analysis of all studies showed that the rate of secondary endovascular reinterventions was smaller in patients treated with the PETTICOAT (*n* = 3 studies; OR, 0.30; 95% CI, 0.10 to 0.94; *p* = 0.04). The results of other postoperative complications (30-day mortality, mortality during follow-up, paraplegia, stroke, and occurrence of endoleak) were lower in the PETTICOAT group but were not statistically significant. The rate of postoperative renal failure was lower in patients treated with the TEVAR (*n* = 4; OR, 1.08; 95% CI, 0.46 to 2.51; *p* = 0.86). *Conclusion*: This meta-analysis suggests that the PETTICOAT procedure is related to the lower rate of secondary endovascular reinterventions for complicated acute, and subacute type B aortic dissections.

## 1. Introduction

Thoracic endovascular aneurysm repair (TEVAR) is widely recognized as a treatment of choice for the management of an array of thoracic aortic pathologies, particularly for acute and complicated type B aortic dissection.

However, recent studies suggest that TEVAR treatment could be complicated by stent-graft-induced new entry (SINE), retrograde type A aortic dissection and enlargement of the residual false lumen, all of which can lead to the development of severe pathological conditions and increased mortality [1]. Over the years, it was noted that TEVAR could contribute to unfavorable distal aortic remodeling, and the concept of using both a covered stent graft and extended bare stents was created. This procedure, named the provisional extension to induce complete attachment (PETTICOAT), has demonstrated a decreased incidence of aorta-related adverse events and fewer reinterventions, with a subsequent impact on the promotion of aorta remodeling [2,3]. However, the number of studies are limited, and it is suggested that the PETTICOAT method should only be used in high-risk patients with complex cases (e.g., multiple reentry tears with complete true lumen collapse) [4]. Recent systemic reviews also do not show an improvement in short- and midterm survival after the PETTICOAT vs. TEVAR [5]. Overall, several meta-analyses conducted before show conflicting results concerning the superiority of the PETTICOAT procedure, without definitive conclusions.

This meta-analysis aimed to investigate the peri- and postoperative complications, as well as the overall mortality during follow-up, after the PETTICOAT vs. TEVAR procedure in acute and subacute type B aortic dissections. The uniqueness of our work lies in its broader perspective of analyzing the adverse effects and complications after both procedures.

## 2. Materials and Methods

### 2.1. Literature Search and Inclusion Criteria

The systematic review was conducted in concordance with the Preferred Reporting Items for Systematic Reviews and Meta-Analyses (PRISMA) statement guidelines [6] and Cochrane Handbook for Systematic Reviews guidelines [7]. The study protocol for this research was not preregistered with a database. We checked the Ovid versions of MEDLINE, PubMed, Embase, and Cochrane Library to recognize studies investigating the occurrence of peri- and postoperative complications and the overall mortality during follow-up after the PETTICOAT vs. TEVAR procedure in acute and subacute type B aortic dissections. The first database search was conducted on 21 February 2022. The following combinations of keywords were used, connected using Boolean operators, to maximize our search sensitivity: “aortic dissection” AND “EVAR” OR “aortic dissection” AND “TEVAR” OR “aortic dissection” AND “endovascular aortic repair” OR “PETTICOAT” OR “bare metal stent” AND “EVAR” OR “bare metal stent” AND “TEVAR” OR “bare metal stent” AND “endovascular aortic repair” OR “bare metal stent” AND “stent graft”. The last database search and the abstract and topic analysis were performed on 22 June 2022, without any language or country of origin limitations; however, only studies published since 2010 were included. The eligibility of the studies was checked by resorting to the PICOS (population, intervention, comparators/controls, outcomes, and study design) question [8], as follows:

1. Population: patients with acute or subacute type B aortic dissections.
2. Intervention: implantation of covered stent grafts + extended bare stents (PETTICOAT) or distally extended PETTICOAT for acute and subacute type B aorta dissections.
3. Comparators/controls: patients who underwent simple TEVAR for type B acute and subacute aorta dissections.
4. Outcomes: the rate of peri- and postoperative complications and overall mortality during follow-up.
5. Study design: observational studies and randomized controlled trials were included.

Reviews, meta-analyses, and case reports were excluded from our research. Studies fulfilling the following criteria were included in the research: more than five patients’ cases, full information on the type of dissection treated and the onset of dissection, as well as reporting clinical and technical outcomes. We studied the perioperative and postoperative complications: 30-day mortality, postoperative stroke, paraplegia, renal failure, mortality during follow-up, surgical or endovascular reintervention, and occurrence of endoleak. The follow-up period for all-cause deaths was 12 months. 

We contacted the authors of two studies to receive more complete information regarding all-cause deaths at 1-year follow-up and the number of patients with acute kidney failure after the TEVAR and PETTICOAT procedures and patients who underwent open surgical reinterventions in each of the two groups [3,9]. We only received a response from the authors of one study [3] and, accordingly, we updated our research with the data. 

The research initiated with studying titles and abstracts consistent with our systematic review. The abstracts that passed the initial screening underwent a full-text review. The suitability of the studies, as compared against our inclusion criteria, was evaluated by three authors independently (N.N., K.K., K.Z.), and potential incongruities were resolved by a fourth author (P.R.). 

### 2.2. Data Extraction and Assessment of Strengths and Weaknesses of Included Studies

**Figure 1:**
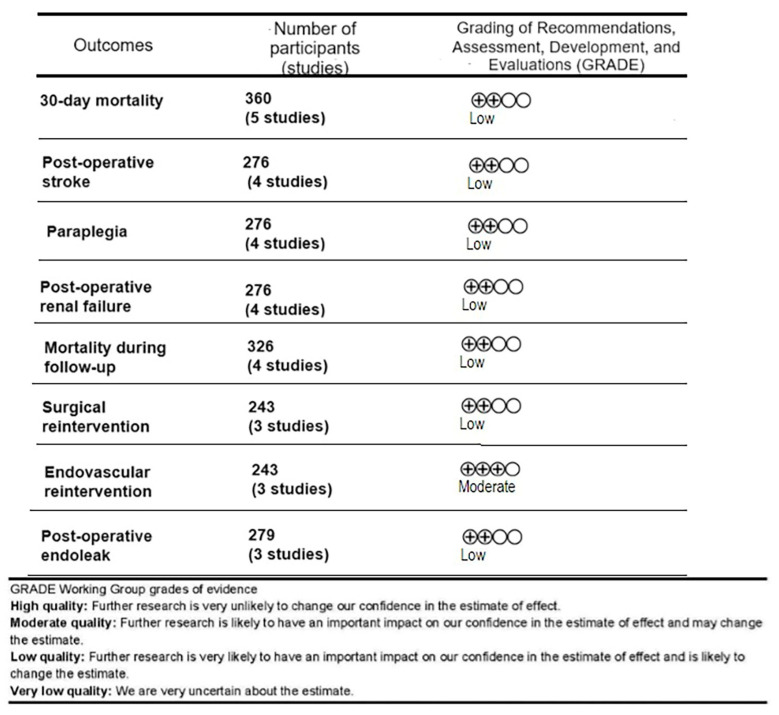
Grading of Recommendations, Assessment, Development, and Evaluations (GRADE score) for each outcome.

Four authors independently (N.N., P.R., K.K., K.Z.) retrieved the data from the studies in predefined tables. The following information was collected: the sample sizes for the PETTICOAT procedure (with subdivision into acute and acute–subacute dissections) and the TEVAR procedure; study design; 30-day mortality; postoperative stroke; the occurrence of postoperative paraplegia; acute renal failure; mortality during follow-up; surgical and endovascular reinterventions (including type I and II endoleaks); occurrence of endoleak. The risk of bias was assessed independently by two authors (N.N. and P.R.). A study assessment tool was taken into consideration to determine the risk of bias in the included studies, which was adapted from a previously reported quality assessment tool and Standard Quality Assessment Criteria for Evaluating Primary Research Papers [10]. Then, the studies were evaluated for a potential risk of bias according to the ROBINS-I [11] and RoB-2 guidelines [12]. Additional criteria significant to this systematic review were evaluated. This included evaluating whether a study provided a clear and objective definition of complicated aortic dissection, which was then a decisive factor in qualifying the patient for the endovascular procedure. An assessment of the technical aspects of the endovascular procedure were also considered (e.g., rate of technical success, devices used, and description of the procedure). We also assessed if the patients’ characteristics were described in each study. We assigned the certainty of the evidence for each outcome according to the GRADE (grading of recommendations, assessment, development, and evaluations) approach (Figure 1).

### 2.3. Statistical Analysis

The PETTICOAT and TEVAR study groups, as well as the mortality and complications rates during follow-up, were retrieved from all papers included in this analysis. These data were used to calculate the odds ratio (OR) for each paper and to summarize them. The ORs are displayed using forest plots. The interstudy heterogeneity was measured with the I2 index, and low, moderate, and high heterogeneity are indicated by values below 25%, between 25% and 75%, and beyond 75%, respectively. Statistical significance was considered when the *p*-value was <0.05. A random effects meta-analysis was performed. The funnel plot was visually inspected for asymmetry to evaluate the publication bias. The analysis was carried out in PQStat (PQStat Software 2022, v.1.8.4. Poznan, Poland).

## 3. Results

### 3.1. Study Identification

After removing the duplicates, the database yielded 2350 studies. Subsequently, 27 studies were included after the initial title and abstract screening. The full-text PDFs of 14 studies were further analyzed against the inclusion criteria. The remaining 10 studies had abstracts in English but full-text PDFs were only available in languages other than English. Nine studies were excluded after the full-text analysis (Table 1). 

**Figure 2:**
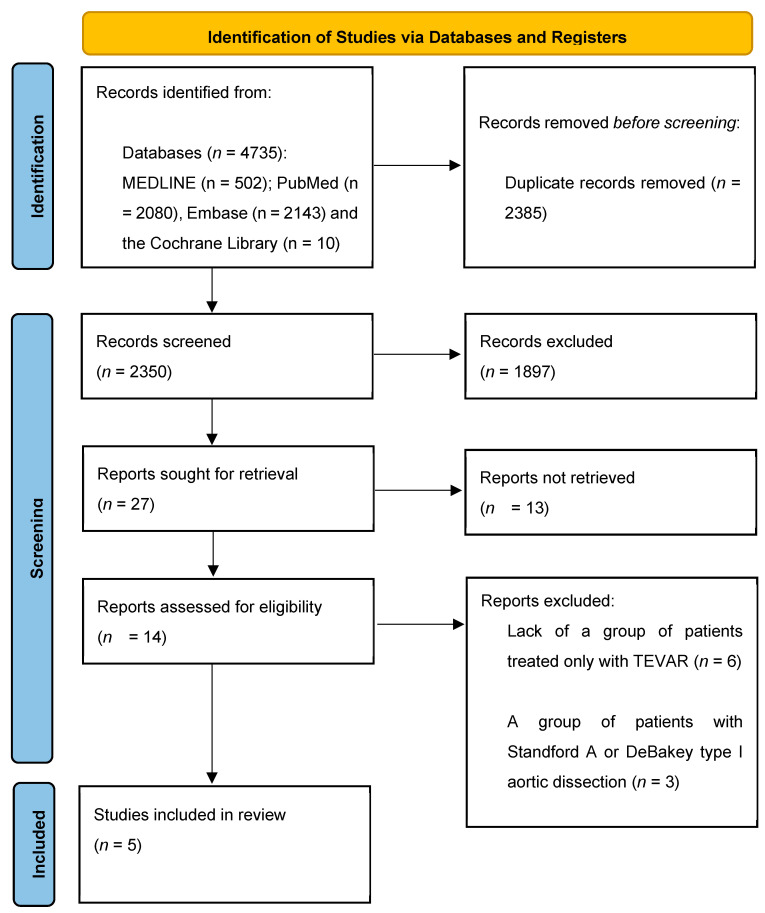
PRISMA 2020 flow diagram for new systematic reviews including only searches of databases only. PRISMA, preferred reporting items for systematic reviews and meta-analyses.

Finally, 5 studies were included in this systematic review [2,3,9,21,22] (Figure 2).

### 3.2. Study Characteristics

A total of 360 patients were included: 143 patients undergoing the PETTICOAT procedure and 217 patients after the TEVAR procedure. In this meta-analysis were included patients with acute and subacute type B aortic dissection, without a chronic type. In two studies, the patients with acute and subacute types constituted one group [2,3], while in the remaining research only patients with acute type B aortic dissection were included [9,21,22]. More detailed information regarding the mean interval time to operation was provided in two studies [2,3]. Patients who underwent conventional TEVAR waited fewer days for an operation; however, in only one study was the result statistically significant [3]. Lin et al. [22] conducted a prospective and randomized study, while the remaining four were retrospective. The sample sizes ranged between 33 and 148. The included studies were conducted between 2018 and 2021. Two studies were performed in Japan [2,3], two in China [21,22], and one in the United States [9]. In all studies, patients with type B aortic dissection were included according to the Standford classification. Patients with uncomplicated aortic dissections were excluded from all studies, as they had received conservative treatment. The definition of “complicated” aortic dissection, qualifying the patient for endografting, differed among the studies. The major indication was rapid aortic expansion [2,3,9,22]. Sultan et al. [9] indicated, as a sufficient criterion, an increase of >3 mm in the aortic diameter within 24 to 72 h. Other studies, however, indicated as important an aortic enlargement > 5 mm from hospital admission, or aortic dilatation > 40 mm [2,3] or aortic expansion ≥ 10 mm while in hospital, or aortic diameter ≥ 60 mm [22]. However, for He et al. [21], an enlarged aortic diameter in the dissected region with evidence of hemothorax and no active contrast extravasation in the computed tomography angiography (CTA) images were included in the definition of complicated aortic dissection. Further details of the inclusion criteria are described in Table 2.

In all studies, patients underwent preoperative assessment of anatomic suitability for endovascular procedure via computed tomography angiography (CTA). The following proximal stent-grafts systems were used: Endurat (Medtronic Cardiovascular, Santa Rosa, CA, USA) [22], Ankura (LifeTech Scientific, Shenzhen, China) [22], Zenith TX2 [2,3,9,21,22] or Hercules (MicroPort, Shanghai, China) [21,22], Relay (Bolton Medical Inc., Sunrise, FL, USA) [3,21], and Talent [21]. The rate of technical success was mentioned in three studies, and for two of them it was 100% for both the PETTICOAT and TEVAR groups [2,3]. He et al. [21] reported primary technical success, defined as an endograft deployment without type I or III endoleak and an absence of open surgical conversion or death within 24 h of operation, in 95.6% of patients in the TEVAR group. The reason for failure in four patients in this group was type IA endoleak, and there was one patient with retrograde acute type A aortic dissection requiring emergent sternotomy. The single technical failure among the PETTICOAT group was due to false lumen (FL) rupture in the abdominal aorta distal to the bare stent. The average operative time was mentioned in only one study and was 50 min and 70 min in the TEVAR and PETTICOAT groups, respectively [21].

**Table 2:** Characteristics of the included studies. cTBAD, complicated type B aortic dissection; TL, true lumen, FL, false lumen; CTA, computed tomography angiography; ICU, intensive care unit.

Study	Country	Study Design	Sample Size	TEVAR Group	PETTICOAT Group	Inclusion Criteria	Exclusion Criteria	Study Endpoints
Hashizume et al., 2020 [3]	Japan	Retrospective cohort study	47	21	26	Patients with acute to subacute cBAD with patent false lumen with rupture, visceral ischemia, rapid aortic enlargement (≥5 mm since hospital admission), and aortic aneurysmal diameter (≥40 mm at symptom onset)	-	Index of TL, index of FL, aorta “remodeling”, technical success, 30-day mortality, in-hospital death, postprocedural: stroke, paraplegia, paraparesis, and endoleak type Ia
He et al. [21]	China	Retrospective cohort study	148	113	35	Patients with acute cTBAD with one or more of the following criteria: branch vessel obstruction, impending rupture (enlarged aortic diameter in the dissected region with evidence of hemothorax and no active contrast extravasation in the CTA), resistant hypertension, persistent pain or symptoms PETTICOAT: clinical evidence of malperfusion and/or radiologic evidence of complete TL collapse	Patients with acute uncomplicated type B aortic dissections	Operative details, technical success, endoleak, retrograde dissection, aortic rupture, stroke, paraparesis or paraplegia, renal failure, bowel ischemia, 30-day and midterm mortality, freedom from reintervention
Matsuoka et al., 2021 [2]	Japan	Retrospective cohort study	48	24	24	Patients with complicated aortic dissections with distal extension into zone 9 or beyond, including both type B and residual type B. Patients with dilatation of the aorta (>40 mm), rapid aortic enlargement (>5 mm during first hospital admission), and impaired perfusion with end-organ ischemia	Patients with hemodynamic instability, traumatic etiology, with limitations of care, chronic aortic dissection for whom the interval to invention was more than 3 months	Primary: aortic remodeling Secondary: aorta-related adverse events (abdominal aneurysmal degeneration, and aortic erosion); success rate of the procedure; paraplegia; stroke; thrombotic events; need for permanent dialysis; 30-day mortality; ICU and hospital lengths of stay; rate of secondary interventions; insufficient aortic remodeling
Lin et al., 2020 [22]	China	Prospective and randomized controlled trial	84	42	42	Patients with acute cTBAD with rapid aortic expansion (≥60 mm or an expansion rate ≥10 mm while in hospital); aortic rupture and/or hypotension/shock; renal or limb ischemia; paraplegia/paraparesis; periaortic hematoma; recurrent or refractory pain; and/or refractory hypertension despite adequate medical therapy	Patients aged <18 years or >80 years, with diagnosed or suspected Marfan syndrome or Standford type A or B aortic dissection with previous placement of thoracic stent-graft; serious bowel malperfusion; and lack of patient’s consent	Primary: all-cause mortality at 5 years after randomization Secondary: endoleak, stent-graft-induced new entry (SINE), aortic rupture, and secondary intervention
Sultan et al., 2018 [9]	United States	Retrospective cohort study	33	12	21	Patients with acute cTBAD with rupture, malperfusion, rapid aortic expansion (an increase of >3 mm in the aortic diameter within 24 to 72 h), and intractable back pain	Patients with connective tissue disorders, sepsis, coagulopathy, previous endovascular aortic repair or TEVAR, or prior open descending thoracic aortic repair PETTICOAT: distal aortic arch radius <35 mm, patient had an unrelated interventional or surgical procedure within 30 days of presentation, and a lack of the patient’s consent	Aortic remodeling, aortic thrombosis, secondary interventions, in-hospital deaths, postoperative stroke, renal failure, length of hospital stay, prolonged respiratory insufficiency, permanent paralysis, and temporary paresis

### 3.3. Patients’ Characteristics

The methods of reporting the patients’ characteristics varied among the studies. The basic characteristics are summarized in Table 3.

Hashizume et al. [3] reported fewer patients’ characteristics in comparison to other studies, providing principally the age and gender of the patients, focusing more on other data, which concerned preprocedural characteristics (e.g., number of entry tears in each zone, aortic dissection acuity, and % emergency). The patients who underwent operation were usually between the 5th and 6th decades of their life. The proportion of men in the TEVAR group and PETTICOAT group were 81 to 92% and 76 to 88%, respectively. There was a high percentage of patients suffering from hypertension both in the TEVAR and PETTICOAT group. In one study, all patients in the TEVAR group had hypertension, and in the PETTICOAT group 41 of 42 suffered from high blood pressure [22]. However, all studies reported no significant differences in the proportions of patients with diabetes, hypertension, coronary artery disease, renal insufficiency, and COPD between the TEVAR and PETTICOAT groups. The proportion of participants who were current or previous smokers ranged from 33% to 67% [9,22], but the results were not statistically significant. Lin et al. [22] mentioned the medical treatment of patients included in the study, and all participants were on calcium-channel blockers, statins, and beta-blockers. The most frequent indications for the TEVAR procedure were organ ischemia and malperfusion [3], refractory chest pain [22], and rapid progression in size [2,9]. The indications for the PETTICOAT were the same, except in one study where more patients with malperfusion without rupture were classified to the PETTICOAT procedure [9]. These results were not statistically significant.

### 3.4. Risk of Bias Assessment

The strengths and weaknesses of the included studies are included in the results in Section 3, together with the forests plots. The risk of bias assessment was calculated accordingly to the ROBINS-I guidelines for the cohort studies [11] and RoB 2 tool for the RCT [12].

Four studies were of a retrospective design [2,3,9,21], and only one was a randomized controlled trial [22]. As a result, certain and important patient factors that could contribute to, for example, secondary reinterventions or early mortality, were not available. In fact, only Lin et al. [22] reported the detailed medical treatment of the patients and the average admission systolic blood pressure and heart rate for each group. The criteria that allowed for patients to undergo endovascular treatment were well defined in all studies (acute or acute–subacute type B aortic dissection with rapid aortic enlargement since hospital admission, dilatation of aorta/pain or symptoms/visceral ischemia) [2,3,9,21,22]. Only one study underlined the specific criteria for distal bare stent placement, which was clinical evidence of malperfusion and/or radiological evidence of complete TL collapse [21]. The reasons for the surgical secondary reinterventions were enlarging dissecting thoracoabdominal aneurysms [3] and persistent malperfusion of vessels that required open surgical fenestrations [21]. The endovascular reinterventions were caused by the remaining type 1 endoleak [2,3] or stent-graft-induced distal re-dissection [21]. Sultan et al. [9] presented the general rate of surgical and endovascular interventions, without the subdivision into the TEVAR and PETTICOAT groups, and, as a consequence, this study was not included in the statistical analysis for the rate of secondary reinterventions, as well as Lin et al. [22], who did not report this variable in their study. The stent-grafts used for the endovascular procedures and operative techniques were well described in all studies. Three-phase CT was used at baseline and at follow-up to assess the changes in the aorta. The mean follow-up ranged between 6 months and even 5 years [22]. For our systematic review, we included the studies that provided results for all-cause mortality at 1-year follow-up.

### 3.5. Data Synthesis

**Figure 3:**
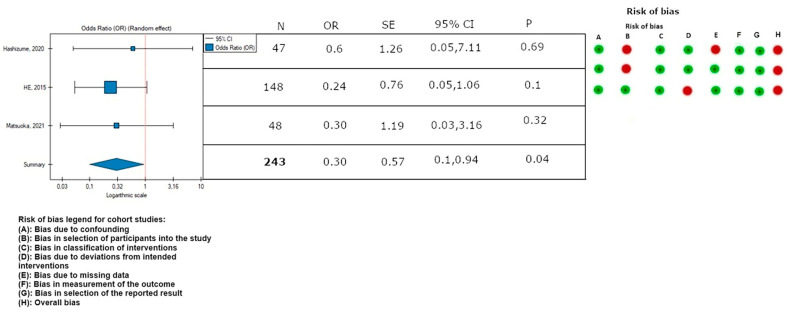
Association between implantation of the TEVAR with a bare metal stent and the rate of endovascular reinterventions, with the rate of bias calculated according to the ROBINS-I guidelines. On the right side of the figure, the green color represents a low risk of calculated bias and the red a moderate risk of bias [2,3,21]. The horizontal lines represent 95% confidence intervals (CIs). N, number of patients; OR, odds ratio; and SE, standard error.

In this meta-analysis, the rate of endovascular reinterventions was lower in patients in the TEVAR with a bare stent (PETTICOAT) group (OR, 0.30; SE, 0.57; 95% CI, 0.10 to 0.94; *p* = 0.04; Figure 3). Low heterogeneity was observed (*I*^2^ = 0%).

**Figure 4:**
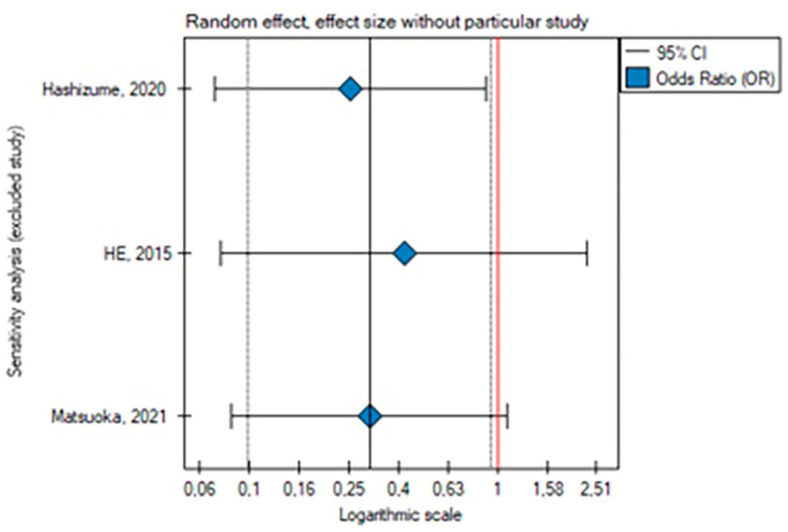
Leave-one-out analysis for the rate of endovascular reinterventions [2,3,21].

**Figure 5:**
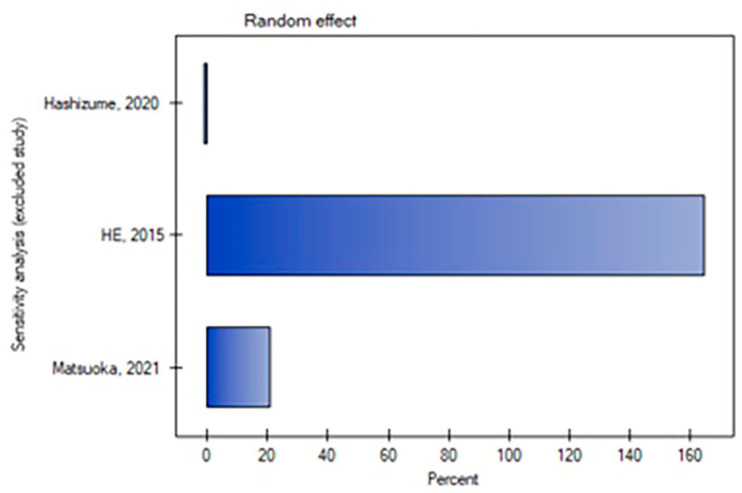
Leave-one-out meta-analysis with random effect and change in precision for the rate of endovascular reinterventions [2,3,21].

The leave-one-out analysis revealed that the result of this meta-analysis for secondary endovascular reinterventions was dependent on the He and Matsuoka study, respectively [2,21] (Figure 4 and Figure 5).

**Figure 6:**
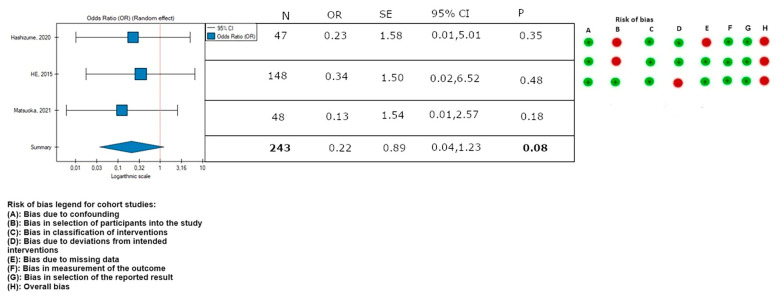
Association between implantation of the TEVAR with a bare metal stent and the rate of surgical reinterventions, with risk of bias calculated according to the ROBINS-I guidelines. On the right side of the figure, the green color represents a low risk of calculated bias and the red a moderate risk of bias [2,3,21]. The horizontal lines represent 95% confidence intervals (CIs). N, number of patients; OR, odds ratio; and SE, standard error.

**Figure 7:**
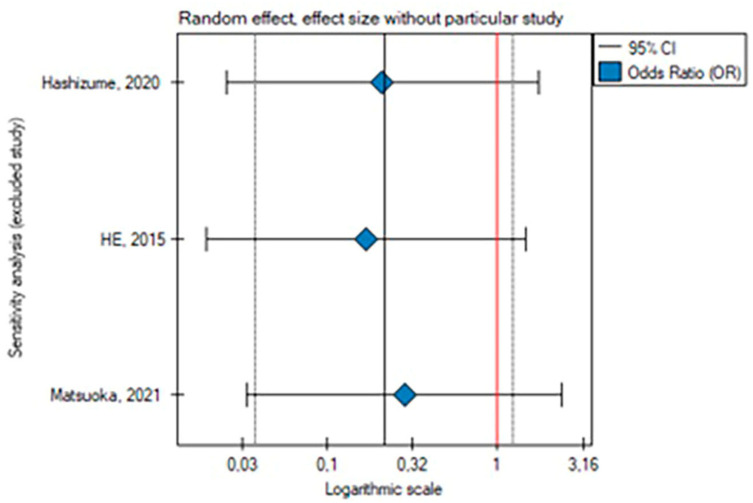
Leave-one-out analysis for the rate of surgical reinterventions [2,3,21].

The rate of surgical reinterventions was lower in patients with the PETTICOAT; however, the results are not statistically significant (OR, 0.22; SE, 0.89; 95% CI, 0.04 to 1.23; *p* = 0.08, *I*^2^ = 0%) (Figure 6 and Figure 7).

**Figure 8:**
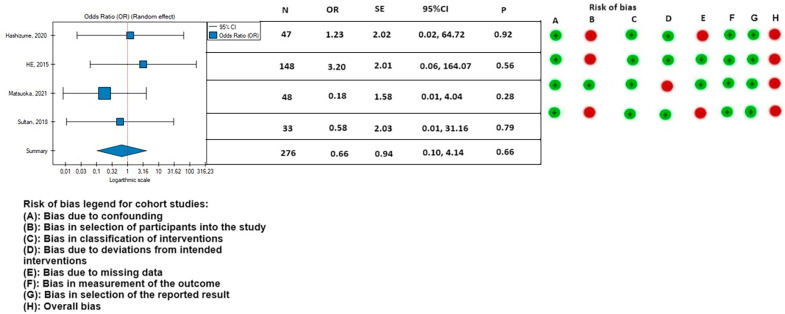
Association between implantation of the TEVAR with a bare metal stent and the rate of postoperative stroke, with the risk of bias calculated according to the ROBINS-I guidelines. On the right side of the figure, the green color represents a low risk of calculated bias and the red a moderate risk of bias [2,3,9,21]. The horizontal lines represents 95% confidence intervals (CI). N, number of patients; OR, odds ratio; and SE, standard error.

**Figure 9:**
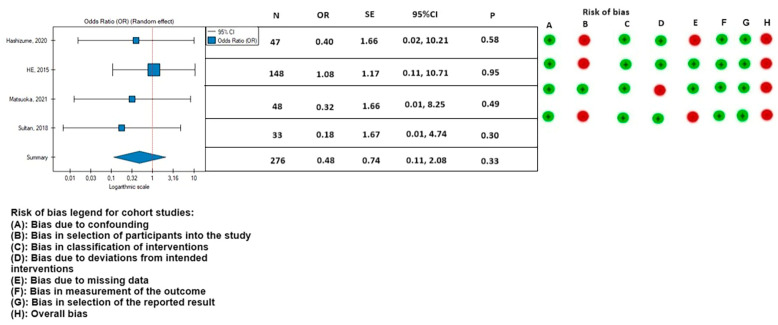
Association between implantation of the TEVAR with a bare metal stent and the rate of paraplegia with the risk of bias calculated according to the ROBINS-I guidelines. On the right side of the figure, the green color represents a low risk of calculated bias and the red a moderate risk of bias [2,3,9,21]. The horizontal lines represents 95% confidence intervals (Cis). N, number of patients; OR, odds ratio; and SE, standard error.

**Figure 10:**
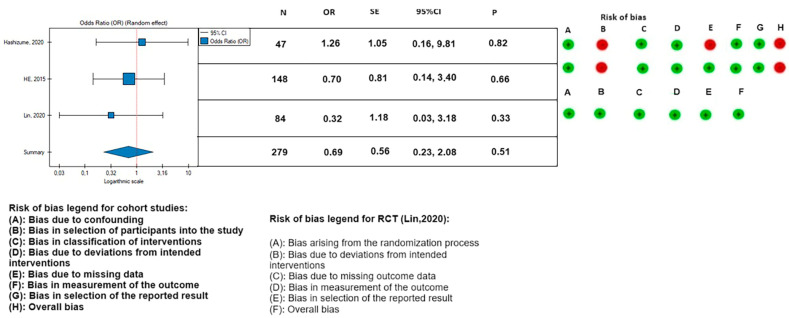
Association between implantation of the TEVAR with a bare metal stent and the occurrence of postoperative endoleak, with the risk of bias calculated for the cohort studies (ROBINS-I) and randomized controlled study (RCT) (RoB2). On the right side of the figure, the green color represents a low risk of calculated bias and the red a moderate risk of bias [3,21,22]. The horizontal line represents 95% confidence intervals (CI). N, number of patients; OR, odds ratio; and SE, standard error.

**Figure 11:**
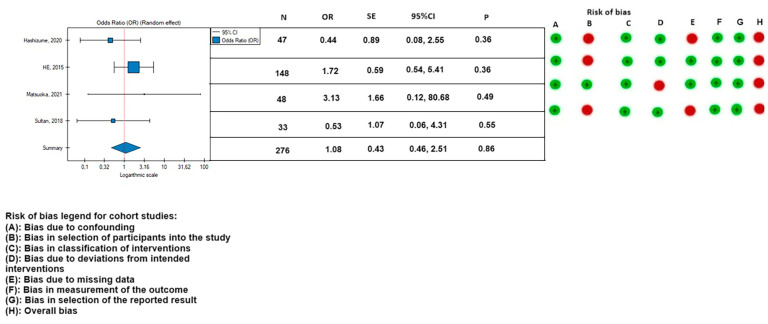
Association between implantation of the TEVAR and postoperative renal failure, with the risk of bias calculated according to the ROBINS-I guidelines. On the right side of the figure, the green color represents a low risk of calculated bias and the red a moderate risk of bias [2,3,9,21]. The horizontal lines represents 95% confidence intervals (CI). N, number of patients; OR, odds ratio; and SE, standard error.

The 30-day mortality appeared to be lower in the PETTICOAT group (OR, 0.46; SE, 0.68; 95% CI, 0.12 to 1.76; *p* = 0.26; *I*^2^ = 0%), as well as the mortality during follow-up (OR, 0.43; SE, 0.54; 95% CI, 0.15 to 1.23; *p* = 0.12; *I*^2^ = 0%), however, the results were not statistically significant. Other postoperative parameters had a lower rate of occurrence in patients treated with the TEVAR with a bare metal stent: rate of postoperative stroke (OR, 0.66; SE, 0.94; 95% CI, 0.10 to 4.14; *p* = 0.66; *I*^2^ = 0%) (Figure 8); paraplegia (OR, 0.48; SE, 0.74; 95% CI, 0.11 to 2.08; *p* = 0.33; *I*^2^ = 0%) (Figure 9); and occurrence of postoperative endoleak (OR, 0.69; SE, 0.56; 95% CI, 0.23 to 2.08; *p* = 0.51; *I*^2^ = 0%) (Figure 10). The rate of postoperative renal failure was lower in patients treated with only TEVAR (OR, 1.08; SE, 0.43; 95% CI, 0.46 to 2.51; *p* = 0.86; *I*^2^ = 0%) (Figure 11).

## 4. Discussion

This meta-analysis suggests that the PETTICOAT procedure is associated with a lower rate of secondary endovascular reinterventions.

Over the past decades, there has been an evolution in endovascular procedures for type B aortic dissections. The concept of using both a covered stent graft and extended bare stents with the intention to promote aortic remodeling with expansion of true lumen was named the PETTICOAT (provisional extension to induce complete attachment). This procedure has demonstrated favorable short-term outcomes and decreased the rate of late complications, including lower rates of endovascular reinterventions compared with conventional endovascular repair [15]. Over the years, few studies have been published that have tested the efficiency of this new endovascular technique. Nishina et al. [23] analyzed the cases of three patients who underwent the full PETTICOAT and confirmed its significance in aortic remodeling; however, a long-term study was not conducted. In a study from 2019, the PETTICOAT was proposed as a selective treatment for patients with multiple distal reentry tears [4], as patients experienced complete false lumen remodeling at the aortoiliac level. Postoperative complications, such as stroke, acute renal failure, paraplegia, or paraparesis were not recorded. It is suggested that extensive aortic coverage is an important risk factor for spinal cord ischemia [24]; however, by now, there are not many studies confirming this hypothesis for the PETTICOAT technique.

The results of some studies are not encouraging, suggesting that the PETTICOAT does not prevent long-term aneurysmal degeneration and, as a consequence, further possible open or endovascular reinterventions [18]. Nevertheless, a recent systematic review analyzed the PETTICOAT and the staged total aortic and branch vessel endovascular (STABILISE) approaches, and both presented a less than 4% perioperative mortality and a high technical success rate [25]. The number of studies on the PETTICOAT is still low, and there is relatively little research on the long-term complications. Further prospective randomized studies could help to establish more reliable conclusions.

There are some limitations of the PETTICOAT technique itself that need to be addressed. The variety of used stent-grafts in studies included in this meta-analysis could certainly affect results. Complications, as an aneurysmal degeneration or re-dissection due to persistent false lumen perfusion, associated with high morbidity and mortality [26], could be directly related to the type of device used or technique of operation. The main concern after endovascular reintervention is an incidence of stent-graft-induced new entry (SINE)m which appears to be grossly reduced in cases treated with the PETTICOAT [3,21]. For every procedure, including endovascular, there is the risk of serious complications during the postoperative period. The complications that resulted in patients’ death 30 days after the TEVAR were abdominal compartment syndrome after abdominal debranch TEVAR, nonocclusive mesenteric ischemia [3], and aortic rupture [21], while in the PETTICOAT group, they included multiple organ failure [3] and aortic rupture [21]. Nevertheless, only one study reported a limb malperfusion, along with radiological pseudocoarctation, which needed the addition of a bare metal stent 2 days after the initial TEVAR [9]. No bowel malperfusion requiring surgery was reported.

Two studies calculated the mean fluoroscopy time which was 22 and 17 min for the TEVAR and 27 and 21 min for the PETTICOAT, respectively [2,21]. Although the times for the PETTICOAT procedure seem to be longer and a higher amount of average contrast volume was used during the procedure [21], the prevalence of acute kidney injury, as well as incidence of permanent dialysis, was not higher in this group of patients compared to the TEVAR group [2,3,9,21].

Although it has been suggested that the PETTICOAT could be associated with lower rates of postoperative adverse events, further studies are needed. The qualification of patients for the procedure, using as a criteriontheir comorbidities, and the use of certain drugs should be conducted further, preferably in randomized studies. The operative details (e.g., devices used and surgeon’s level of experience) and the procedure itself should be equal for all patients.

The follow-ups differed among the studies, ranging from an average of 6 months [9] to 1 year [3,21], 2 years [2] and even 5 years [22]. In this meta-analysis, we report a low heterogeneity, even though the methodologies among the included studies was quite different. The main limitation of our meta-analysis was the relatively few studies included, only one of which is a randomized prospective trial [22]. As this systematic review was not registered in the PROSPERO database, and the number of studies included in this research is limited (n < 10), this situation could have contributed to an inappropriate estimation of the publication bias.

The high homogeneity of this meta-analysis suggests that the PETTICOAT intervention for complicated acute, and subacute type B aortic dissections is correlated with lower rates of secondary endovascular reinterventions. The PETTICOAT seems to be a promising treatment for patients with aortic dissection, which could potentially save them from further reinterventions. However, more studies, preferably well-designed randomized controlled trials, are needed to prove the effectiveness of the PETTICOAT compared to the TEVAR.

## 5. Conclusions

The results of this study suggest that patients with complicated acute, and subacute type B aortic dissection treated with the PETTICOAT technique have a lower rate of secondary endovascular reinterventions. Further prospective studies are needed to clarify this correlation.

## Figures and Tables

**Table 1:** Details of excluded studies.

Study	Reason for Exclusion
Molinari et al. (2019) [4]	Lack of group of patients treated with TEVAR, and the study included only 4 cases.
Hsu et al. (2015) [13]	Included patients with DeBakey type I aortic dissection.
Huang et al. (2018) [14]	Included patients with DeBakey type I aortic dissection.
Hofferberth et al. (2012) [15]	Included patients with Standford type A aortic dissection.
Leo et al. (2021) [16]	Lack of a group of patients treated with TEVAR.
Hsu et al. (2021) [17]	Lack of a group of patients treated with TEVAR.
Mascia et al. (2021) [18]	Lack of a separate group of patients treated only with TEVAR.
Lombardi et al. (2018) [19]	Lack of a separate group of patients treated only with TEVAR.
Melissano et al. (2012) [20]	Lack of a separate group of patients treated only with TEVAR.

**Table 3:** Patients’ characteristics. COPD, chronic obstructive pulmonary disease.

	TEVAR Group	PETTICOAT Group
Hashizume et al., 2020 [3]		
Age (y)	58.3	56.0
Male sex (in %)	84.6	81
He et al., 2015 [21]		
Age (y)	43	42
Male sex (in %)	81.4	85.7
Hypertension (in %)	97.3	97.1
Diabetes (in %)	8.8	5.7
Coronary artery disease (in %)	11.5	17.1
COPD (in %)	8.8	11.4
Renal insufficiency (in %)	6.2	5.7
Matsuoka et al., 2021 [2]		
Age (y)	60	53
Male sex (in %)	83	79
Hypertension (in %)	83	83
Diabetes (in%)	4	8
Coronary artery disease (in %)	8	0
Renal insufficiency (in %)	33	42
Lin et al., 2020 [22]		
Age (y)	52.1	55.1
Male sex (in %)	88.1	88.1
Hypertension (in %)	100	97.6
Smoking (in %)	30.9	33.3
Diabetes (in %)	16.7	14.3
Coronary artery disease (in %)	4.8	9.5
COPD (in %)	9.5	4.8
	Sultan et al., 2018 [9]	
Age (y)	62.6	64.5
Male sex (in %)	92	76
Hypertension (in %)	75	86
Smoking (in %)	67	57
Renal insufficiency (in %)	0	5

## Data Availability

The datasets analyzed during the current study are available from the corresponding author upon reasonable request.
